# PD-L1 Expression and CD8^+^ T Cell Infiltration Predict a Favorable Prognosis in Advanced Gastric Cancer

**DOI:** 10.1155/2018/4180517

**Published:** 2018-05-29

**Authors:** Yangyang Wang, Chunchao Zhu, Wei Song, Jun Li, Gang Zhao, Hui Cao

**Affiliations:** ^1^Department of Gastrointestinal Surgery, Renji Hospital, School of Medicine, Shanghai Jiao Tong University, Shanghai 200127, China; ^2^State Key Laboratory of Oncogenes and Related Genes, Shanghai Cancer Institute, Renji Hospital, School of Medicine, Shanghai Jiao Tong University, Shanghai 200240, China; ^3^Department of Surgery, Quzhou Women & Children Hospital, Quzhou 324000, China

## Abstract

Advanced gastric cancer (AGC) has high morbidity and mortality in East Asia, and it is urgent to explore new treatments to improve patient prognosis. Programmed death-1 (PD-1)/programmed death-ligand 1 (PD-L1) inhibitors have exhibited remarkable activity in clinical trials and were approved by the FDA for clinical therapy in several types of tumors. Here, we evaluated PD-L1 expression and T cell infiltration in AGC. Positive tumor PD-L1 expression was detected in 171 AGCs (33.60%) out of 509 AGCs. PD-L1 expression was positively correlated with CD8^+^ T cell infiltration. Then, PD-L1 and CD8A mRNA expression was analyzed using gastric cancer data from the TCGA database, confirming a positive correlation. Patient survival was assessed according to PD-L1 status and the T cell infiltration density. PD-L1 expression and a high density of CD8^+^ T cells in AGCs were associated with improved prognosis, whereas no significant difference was noted between PD-1 and CD3 expression. In contrast, a high density of FOXP3^+^ T cells in AGCs indicated a poor prognosis. Multivariate Cox regression analysis revealed that CD8^+^ T cell density acts as an independent predictor of overall survival (OS) in AGC patients. Taken together, this study further highlights targets for immune checkpoint-based therapy in AGC.

## 1. Introduction

Gastric cancer (GC) is the fifth most common malignancy and the third leading cause of cancer-related death worldwide [1]. The recent CONCORD-3 study was published in *Lancet* this year. The worldwide surveillance of cancer survival reported in this study demonstrated that GC ranked second in cancer incidence in China with lung cancer ranking first (GC, 15.6%; lung cancer, 22.6%) [2]. Currently, surgical resection and perioperative chemotherapy are routine treatments for AGC. The prognosis of this cancer is dismal, and the need for new strategies to treat AGC is pressing.

Immunotherapy, especially immune checkpoint blockade, has emerged as a promising cancer treatment [3]. Immune checkpoint inhibitors, such as anti-PD-1 (nivolumab and pembrolizumab), anti-PD-L1 (atezolizumab), and anti-CTLA-4 (ipilimumab) drugs, were approved by the Food and Drug Administration (FDA) to treat various types of cancer [4, 5]. The above inhibitors were recommended by the FDA for the treatment of melanoma, non-small-cell lung cancer, and other cancers. Regarding GC therapy with immune checkpoint inhibitors, although no drug has been recommended by the FDA, several clinical trials revealed survival benefits after anti-PD-1 treatment [6, 7].

PD-L1 expression in tumor cells and the immune checkpoint blockade therapeutic response have a close relationship. In a previous study involving 17 patients with PD-L1-negative tumors, none of the patients exhibited an objective response, while for PD-L1-positive tumors, 9 of 25 patients (36%) exhibited an objective response [8]. In another study involving patients with recurrent or metastatic PD-L1-positive gastric cancer, the anti-PD-1 drug pembrolizumab exhibited a promising antitumor effect [7]. Mismatch repair deficiency can also indicate the response of immune checkpoint blockage, and a large proportion of mutant neoantigens in mismatch repair-deficient cancers make them sensitive to anti-PD-1 therapy [9]. PD-L1 expression was significantly associated with mismatch repair deficiency in a large number of patients representing several tumor types [10, 11]. Based on the above data, we concluded that tumor PD-L1 expression status in cancer played an important role in the immune microenvironment. However, studies on tumor PD-L1 expression in GC with large patient numbers are needed to dissect the detailed mechanism.

We investigated PD-L1 expression and T cell infiltration in a tumor microarray (TMA) representing 509 AGC patients. The correlation between PD-L1 expression and T cell infiltration was examined in our study and using GC data from the TCGA database. Finally, the relationships between PD-L1 status and T cell infiltration with patient overall survival (OS) were analyzed.

## 2. Materials and Methods

### 2.1. Patients and TMA Construction

This is a retrospective analysis of 509 patients with primary gastric cancer who underwent gastrectomy at the Department of Gastrointestinal Surgery, Renji Hospital, School of Medicine, Shanghai Jiao Tong University, from January 2006 to December 2011. The final follow-up date was December 31, 2017, for all cases examined. OS time was defined as the interval between the gastrectomy and patient death or survival. A total of 242 mortalities occurred, which were all due to cancer-associated causes. All patients received the standard treatments such as D2 radical resection and first-line adjuvant chemotherapy according to the NCCN guide. Only 25 patients did not finish the standard chemotherapy for their personal reasons or inability to tolerate side effects. There was no difference in the number of not finishing standard chemotherapy between PD-L1-positive and -negative groups. We excluded the following types of patients: (1) patients with recurrent gastric cancer after the radical operation, (2) patients receiving neoadjuvant chemotherapy or previous radiotherapy, (3) patients suffering from other malignant tumors, and (4) patients with autoimmune or immunodeficiency diseases.

We collected formalin-fixed paraffin-embedded (FFPE) tissue blocks from the pathology department of Renji hospital. Tumor TNM stage was assigned based on pathological tumor, node, and metastasis staging per the American Joint Committee on Cancer (AJCC 8th edition) staging system. For each case, the diagnosis was confirmed by two senior pathologists through a review of H&E-stained slides. Representative FFPE blocks were chosen to punch onto glass slides to construct the TMA. Every patient's tumor tissue on the TMA was consecutive, and the TMA was constructed using a tissue arrayer with 5 *μ*m thickness.

This study was approved by the ethics committee of Renji Hospital, Shanghai Jiao Tong University School of Medicine, for the use of samples. Informed consent was obtained from all enrolled patients before study inclusion.

### 2.2. Immunohistochemistry

Immunohistochemistry (IHC) was performed on the TMA using antibodies specific to PD-L1 (1 : 100, Abcam, UK, ab205921), PD-1 (1 : 100, CST, USA, 43248), CD3 (1 : 200, Wuhan Goodbio Technology Co., Ltd., China), CD8 (1 : 100, Wuhan Goodbio Technology Co., Ltd., China), and FOXP3 (1 : 200, CST, USA, 98377). Briefly, after tissue sections were deparaffinized, rehydrated with graded ethanol, incubated with 0.3% hydrogen peroxide for 30 minutes, and blocked with 10% BSA (Sangon, Shanghai, China), slides were first incubated using the antibody at 4°C overnight and then labeled with the HRP second antibody (Thermo Scientific, US) at room temperature for 1 h. Positive staining was visualized with DAB substrate liquid (Gene Tech, Shanghai) and counterstained with hematoxylin [12]. All the sections were observed and photographed with a microscope (Carl Zeiss, Germany). In the following analysis, we excluded immune cells in vessels, lymph nodes and lymphatics, necrotic tissue, or necrosis-adjacent areas.

### 2.3. IHC Evaluation

Tumor PD-L1 expression in the cytoplasm and membrane of tumor cells was evaluated based on immunostaining. The PD-L1-positive group was defined based on greater than 5% of stained cells regardless of cytoplasmic or membrane staining. The remaining cases comprised the negative group. The criterion for classification in the PD-1 high expression group was greater than 5 cells stained per high-power field (HPF), whereas the remaining cases comprised the low expression group [13]. We chose four random areas (amplification 200x, 0.34 mm^2^) on the TMA for each case and counted the average CD3^+^, CD8^+^, and FOXP3^+^ cell density. According to the median number of stained cells (CD3, 80/0.34 mm^2^; CD8, 35/0.34 mm^2^), patients were dichotomized into the high and low density group. To evaluate Foxp3^+^ T cells, given that few cells were stained, we defined the high infiltration group as greater than 5 stained cells/HPF, whereas the remaining cases comprised the low infiltration group. Digital image analysis and Nikon DR-Si2 cell count software were used for the staining evaluation described above, and the results were verified by two senior pathologists who were blinded to the clinicopathological data.

### 2.4. TCGA Database Analysis

From 450 GC samples from the TCGA database, we selected 415 tumor tissue samples except 35 normal tissue samples. We analyzed the correlation between PD-L1 and CD8A expression at the mRNA level.

### 2.5. Statistical Analysis

Standard statistical tests were used to analyze clinical data. Associations between PD-L1 expression and clinicopathological factors were tested using a *χ* ^2^ test or Fisher's exact test. The correlation between PD-L1 and CD8A mRNA expression was calculated using the Spearman correlation test. Survival analysis was performed using the Kaplan-Meier method and the long-rank test. Univariate and multivariate analysis were conducted using the Cox proportional hazards model to analyze prognostic factors. All statistical tests were 2-sided, and *P* < 0.05 was considered statistically significant. All statistical analyses were performed using SPSS 16.0 statistical package software (SPSS, Chicago, IL, USA) or GraphPad Prism (GraphPad Software Inc., San Diego, CA).

## 3. Results

### 3.1. Clinicopathological Findings

A retrospective cohort study of 509 AGC patients, including 49 TNM stage I cases, 172 TNM stage II cases, and 288 TNM stage III cases, was conducted. The median age of the AGC patients was 62 (22–89) years, and the median OS time was 48 (2–117) months. A total of 242 (47.54%) patients died during the follow-up period. Among the 509 cases, male patients (347/509) and a low position of the lesion (360/509) represented a large proportion of the cohort. In total, 96 and 80 cases exhibited blood vessel and perineuronal invasion, respectively, among the 509 AGC patients. The detailed clinicopathological characteristics of the patients are presented in Table 1.

### 3.2. PD-L1 Expression in AGC and Its Association with Clinicopathological Parameters

**Figure 1:**
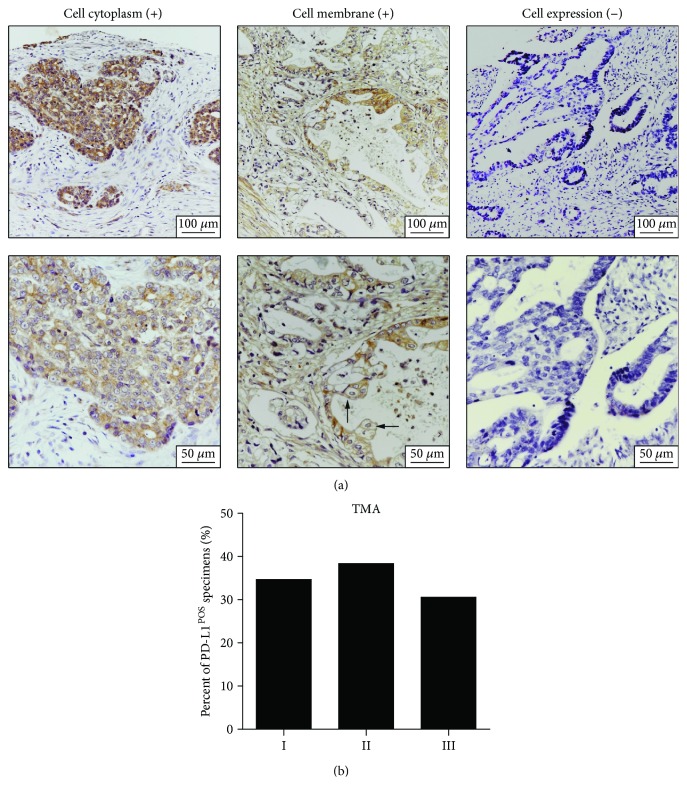
PD-L1 expression in AGCs. (a) Representative samples of IHC staining of PD-L1, including tumor cell cytoplasm staining, tumor cell membrane staining, and negative control, are presented. The lower panel (400x original magnification) is the zoom-in image of the upper panel (200x original magnification). The arrows indicate the membrane PD-L1 expression in tumor cells. (b) The percent of tumor PD-L1-positive specimens in TNM stage I, II, and III GC patients.

In tumor cells, PD-L1 is expressed in the cytoplasm and on membranes (Figure 1(a)). Tumor PD-L1 expression was detected in 171 (33.60%) cases among 509 AGC patients. Regarding TNM stage I, II, and III patients, the percent of tumor PD-L1-positive patients among the three stages did not differ significantly (*P* = 0.2255) (Figure 1(b)).

The relationship between tumor PD-L1 expression and the clinical characteristics of AGC patients is presented in Table 1. PD-L1 expression was positively associated with tumor length-diameter (*P* = 0.0045). No significant relationship was found between tumor PD-L1-positive status and other clinicopathological features.

### 3.3. T Cell Infiltration in Tumor Tissues and Its Association with Clinicopathological Parameters

**Figure 2:**
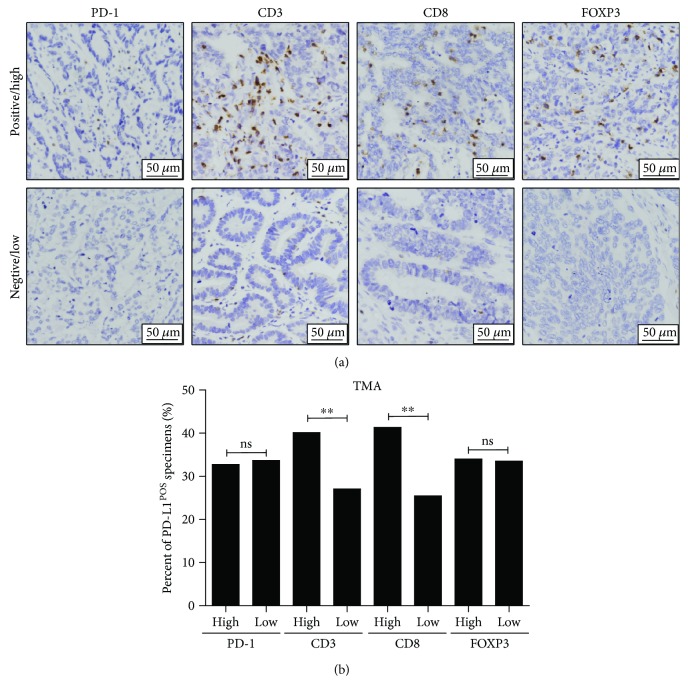
PD-1^+^ immune cell and T cell infiltration in AGCs. (a) Representative samples of IHC staining of PD-1, CD3, CD8, and FOXP3, including high and low infiltration for each marker, are presented. (b) The percent of tumor PD-L1-positive specimens in the high and low expression groups of PD-1, CD3, CD8, and FOXP3. ^∗∗^ *P* < 0.01; ns: no statistical significance.

PD-1 expression was present in infiltrating immune cells, and CD3^+^, CD8^+^, and FOXP3^+^ T cell infiltration in tumor tissues was evident (Figure 2(a)). Regarding the assessments of PD-1 expression and clinicopathological characteristics, we found that PD-1 expression exhibited a close relationship with perineuronal invasion (*P* = 0.0241). No close relationship between PD-1 expression and other clinicopathological features was found (Supplementary Table S1). Regarding the T cell infiltration and patient clinicopathological characteristics analysis, high CD3^+^ T cell infiltration in tumor tissues was positively associated with the patient's Lauren type (*P* = 0.0243). CD8^+^ T cell infiltration in tumor tissues exhibited a close relationship with lymph node metastasis (*P* = 0.0242) (Supplementary Table S2 and S3). No significant relationship was noted between FOXP3^+^ T cell infiltration and any clinicopathological feature (Supplementary Table S4).

### 3.4. Association of PD-L1 Expression with T Cell Infiltration

**Figure 3:**
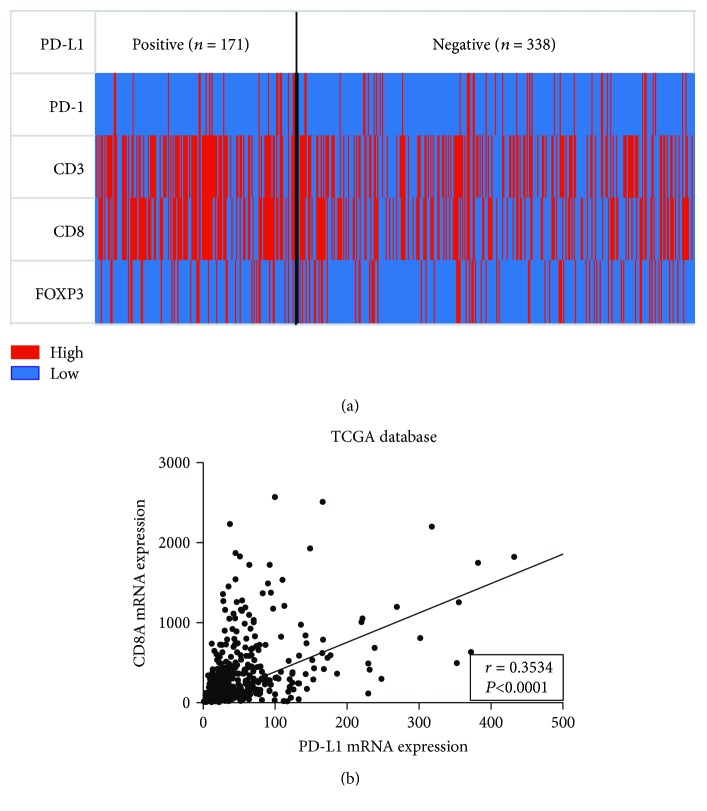
The relationship between PD-L1 expression and T cell infiltration in 509 AGC patients and TCGA database. (a) The heat map of PD-1, CD3, CD8, and FOXP3 high and low infiltration in tumors of PD-L1-positive and -negative AGC patients. (b) The correlation between PD-L1 and CD8A mRNA expression levels in GC patients in the TCGA database.

No significant difference in the number of PD-L1-positive patients was noted between the PD-1 high and low expression groups (*P* = 0.8860). Significant differences in the number of PD-L1-positive patients were noted between the CD3^+^ and CD8^+^ T cell high and low infiltration groups (*P* = 0.0018 and *P* = 0.0001, resp.). Regarding FOXP3, no significant difference in PD-L1-positive patients was noted between the high and low expression groups (*P* = 0.9215) (Figure 2(b)). We constructed a heat map to analyze PD-L1 expression based on T cell tumor infiltration and found that the percentages of CD3^+^ and CD8^+^ T cell high infiltration patients in the PD-L1-positive group were increased compared with the PD-L1-negative group (Figure 3(a)). Next, the association between PD-L1 and CD8A expression at the mRNA level was analyzed using GC data from TCGA database, and a positive correlation was noted (*r* = 0.3534, *P* < 0.0001) (Figure 3(b)).

### 3.5. PD-L1 Expression and T Cell Infiltration Is Associated with Patient OS

**Figure 4:**
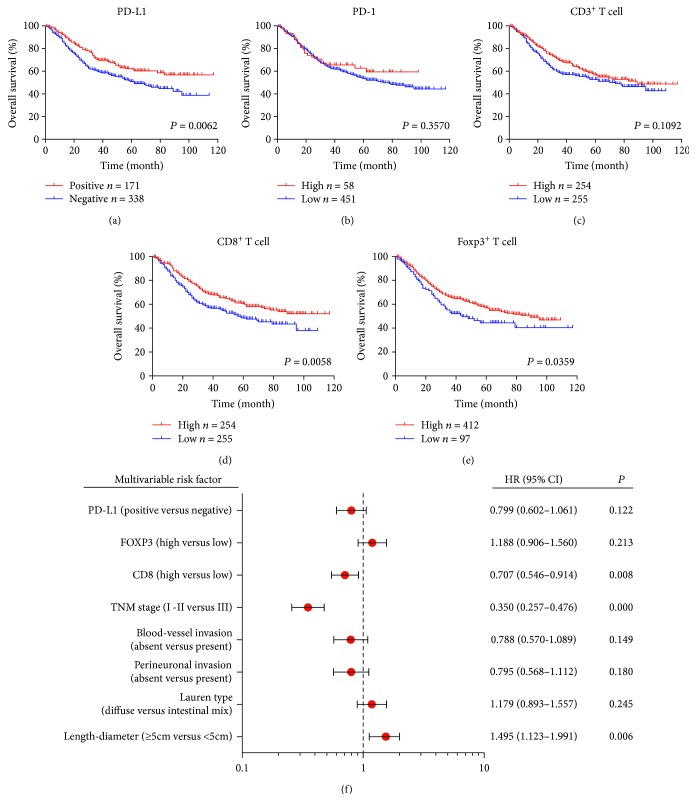
Prognostic value of tumor PD-L1 expression and T cell infiltration in AGC patients. (a) Kaplan-Meier survival curves for OS based on PD-L1, PD-1, CD3, CD8, and FOXP3 status. (b) After univariate analysis, we selected statistically significant risk factors, including PD-L1, FOXP3, CD8, and other clinicopathological parameters, for multivariable analysis.

Kaplan-Meier analysis was performed to evaluate OS according to PD-L1, PD-1, CD3, CD8, and FOXP3 expression in AGC tumors (Figures 4(a)–4(e)). Positive tumor PD-L1 expression and high CD8^+^ T cell infiltration were associated with improved OS compared with negative expression or the low infiltration group (*P* = 0.0062 and *P* = 0.0058, resp.) (Figures 4(a) and 4(d)). Conversely, high FOXP3^+^ T cell infiltration was associated with worse OS than low infiltration (*P* = 0.0359) (Figure 4). No significant differences were noted between the high and low PD-1 and CD3 expression groups (*P* = 0.3570 and *P* = 0.1092) (Figures 4(b) and 4(c)).

### 3.6. Univariate and Multivariable Analysis of Prognostic Parameters for Survival in AGC Patients

Characteristics, including PD-L1 status, T cell infiltration density, and clinicopathological features, were analyzed using Cox proportional hazards regression models to assess the prognostic values (Table 2 and Figure 4(f)). In the univariate analysis of AGC patients, tumor PD-L1-positive status (HR = 0.668, 95% CI: 0.505–0.885, *P* = 0.005), high CD8^+^ T cell infiltration in tumor (HR = 0.691, 95% CI: 0.536–0.891, *P* = 0.004), and high FOXP3^+^ T cell infiltration in tumor (HR = 1.434, 95% CI: 1.061–1.938, *P* = 0.019) were revealed as protective or risk factors for OS in AGC patients. Next, we selected potential prognostic factors based on univariate results (*P* < 0.05) to conduct multivariable analysis. High CD8^+^ T cell infiltration in tumor (HR = 0.707, 95% CI: 0.546–0.914, *P* = 0.008), TNM stage (HR = 0.350, 95% CI: 0.257–0.476, *P* ≤ 0.001), and length-diameter (HR = 1.495, 95% CI: 1.123–1.991, *P* = 0.006) could act independent predictors of OS for AGC patients. However, tumor PD-L1-positive status (HR = 0.799, 95% CI: 0.602–1.061, *P* = 0.122) and high FOXP3^+^ T cell infiltration in tumor (HR = 1.188, 95% CI: 0.906–1.560, *P* = 0.213) were not independent predictors for AGC prognosis. Other clinicopathological parameters, including blood vessel invasion (HR = 0.788, 95% CI: 0.570–1.089, *P* = 0.149), perineuronal invasion (HR = 0.795, 95% CI: 0.568–1.112, *P* = 0.180), and Lauren type (HR = 1.179, 95% CI: 0.893–1.557, *P* = 0.245) exhibited no significant differences in the multivariable analysis (Figure 4(f)).

## 4. Discussion

Gastric cancer, especially at an advanced stage, has limited therapeutic options. The majority of patients are diagnosed at an advanced stage in China as gastroscopy is not as commonly applied in China as in other developed countries. Current conventional treatments for gastric cancer include surgery and perioperative chemotherapy [14]. Immunotherapy, especially immune checkpoint inhibitors, may provide a new opportunity for the treatment of gastric cancer in the future as they have succeeded in the treatment of other solid tumors. In this context, we conducted this study to characterize the roles of PD-L1 and the immune microenvironment in GC patients.

PD-L1, which is also named CD274 or B7H1, is one ligand of PD-1 that is expressed on various types of tumor cells [15]. PD-L1-positive tumors may indicate immune-active tumors that can respond to anti-PD-1 and/or PD-L1 therapies [16]. PD-L1 interaction with its receptor, PD-1, impairs T cell activation and cytokine production. During infection or inflammation in normal tissue, this interaction plays an important role in preventing autoimmunity during the immune response by maintaining homeostasis. In the tumor microenvironment, PD-L1 and PD-1 interaction imparts tumor immunity evasion by inactivating cytotoxic T lymphocytes (CTLs). Previous studies have reported that PD-L1 expression in tumor cells can act as a prognostic factor in various human malignancies, but the conclusion was not consistent even among the same type of tumors. This controversy underscores the importance of our study assessing PD-L1 status in the prognosis of GC patients.

By analyzing the proportion of PD-L1-positive patients among GC patients at different TNM stages, we observed no significant differences. A previous study on PD-L1 expression in non-small-cell lung cancer (NSCLC) demonstrated that high PD-L1 expression was associated with younger patient age and high tumor grade. No associations with sex, tumor size, stage, nodal status, EGFR, or KRAS mutation status were noted. PD-L1 expression appeared to be a favorable prognostic factor in early-stage disease, and the results differed for advanced-stage patients [17]. In our study, we analyzed tumor length-diameter and PD-L1-positive status. We found that the small tumor size (<5 cm) group contained a larger percent of PD-L1-positive GC patients. Other clinicopathological parameters showed no differences in the PD-L1-positive and PD-L1-negative groups.

High PD-L1 expression was associated with high CD8^+^ T cell infiltration in a pancancer analysis study. The results demonstrated that PD-L1 expression exhibited a positive correlation with CD8A expression. Patients exhibiting high or low expression of both proteins were increased compared with patients with high or low expression of only one protein [18]. In our study analysis, we also found that PD-L1-positive status exhibited a tight relationship with CD3^+^ or CD8^+^ T cell infiltration given that the PD-L1 high expression group contained a larger percent of CD3^+^ or CD8^+^ T high infiltration patients than did the low expression group. GC analysis of the PD-L1 and CD8A mRNA expression levels reported in TCGA database revealed a positive correlation between PD-L1 and CD8A mRNA. Another study that classified melanoma tumors based on T cell infiltration and PD-L1 expression demonstrated that 38% were type I cancers (PD-L1^+^TIL^+^), 41% were type II cancers (PD-L1^−^TIL^−^), 1% were type III cancers (PD-L1^+^TIL^−^), and 20% were type IV cancers (PD-L1^−^ TIL^+^) [19]. PD-L1 expression exhibited a positive correlation with CD8^+^ T cell infiltration. This result was consistent with that of our study and another study of synovial sarcoma [20].

Next, we analyzed PD-L1 expression based on GC patient survival. Patients with high PD-L1 expression exhibited prolonged OS times compared with the low expression group. In fact, high PD-L1 expression was associated with a better prognosis than low PD-L1 expression in several cancer types, including gastric cancer [21], colorectal cancer [22, 23], breast cancer [24, 25], metastatic melanoma [26], Merkel cell carcinoma [27], glioblastoma [28], and other cancer [29–31]. Other studies reported that PD-L1 expression status is associated with poor prognosis. PD-L1 expression is upregulated in multiple human cancers and attenuates the antitumor immune response [32–35].

Two mechanisms of the upregulation of PD-L1, including the innate immune response and adaptive immune response, have been proposed. The innate immune response leads to PD-L1 upregulation due to dysregulated oncogenic signaling pathways and chromosomal alterations and amplifications in the tumor. In the adaptive immune response, tumor-infiltrating cytotoxic T lymphocytes (CTLs) secrete IFN-*γ* when they encounter tumor antigens, causing an adaptive response to IFN-*γ* and leading to the upregulation of PD-L1 in tumor cells. The induced expression of PD-L1 in the tumor microenvironment creates a “shield” to avoid attack from activated effector T cells. Consequently, under these circumstances, PD-L1 expression is considered a marker of an active host antitumor immune response [16]. It is not contradictory that high PD-L1 expression levels are associated with better clinical outcomes for patients with an activated immune status.

Based on the above analysis, PD-L1 expression associated with tumor-infiltrating immune cells was a positive prognostic feature. In a study to determine whether tumor-infiltrating lymphocytes (TILs) can predict the clinical prognosis in gastric cancer, the densities of CD3^+^ and CD8^+^ TILs remained independent prognostic factors in multivariate survival analysis [36]. In our study, a high density of CD8^+^ T cell tumor infiltrate indicated an improved prognosis compared with the low density group, and CD8^+^ T cell infiltration was an independent prognostic factor in the multivariate survival analysis. In CD3^+^ T cell infiltration analysis, no significant difference was noted between GC patients with high and low infiltration. It is possible that CD3^+^ T cells contained various T cell types with different functions in the immune response. CD8^+^ T cells act as a type of immune cell that directly kills or eliminate tumor cells in the tumor microenvironment. Conversely, FOXP3^+^ T cells can suppress antitumor immunity. Next, FOXP3^+^ T cell high and low tumor infiltration were analyzed based on OS. We found that a higher density of FOXP3^+^ T cell tumor infiltration was associated with a worse survival. This result was consistent with a previous study in other tumors [37].

## 5. Conclusions

In our study, we explored the relationship between PD-L1 expression and T cell tumor infiltration among 509 AGC patients. We found that PD-L1-positive status was correlated with high CD3^+^ and CD8^+^ T cell infiltration. PD-L1 and CD8A mRNA expression levels were positively correlated among GC patients in the TCGA database. Positive PD-L1 tumor expression and a high density of CD8^+^ T cells in AGCs were both associated with increased OS time, whereas no significant differences were noted in the PD-1 and CD3 high and low groups. In contrast, a high density of FOXP3^+^ T cell infiltration was associated with poor prognosis. Multivariate Cox regression analysis revealed that CD8^+^ T cell density could act as an independent predictor of OS in AGC patients. Taken together, positive tumor PD-L1 expression and high CD8^+^ T cell infiltration might have implications for targeting the PD-L1/PD-1 axis and the treatment of GCs. The prognostic value and immune pattern might be useful for guiding treatment in the future.

## Figures and Tables

**Table 1:** Correlation between tumor PD-L1 expression and clinicopathological parameters in GC patients.

Clinicopathological features	Cases	PD-L1 expression		*P* value (*χ* ^2^ test)
		Positive	Negative	
		171	338	
Gender				
Male	347	116	231	0.9077
Female	162	55	107	
Age(years)				
≤60	224	65	159	0.0526
>60	285	106	179	
Tumor location				
Up	73	22	51	0.8970
Middle	105	35	70	
Low	260	89	171	
Total	66	24	42	
Remnant	5	1	4	
Length-diameter				
<5 cm	232	93	139	0.0045^∗∗^
≥5 cm	277	78	199	
Lauren type				
Intestinal	163	62	101	0.3268
Diffuse	326	102	224	
Mix	20	7	13	
Blood vessel invasion				
Absent	413	139	274	0.9519
Present	96	32	64	
Perineuronal invasion				
Absent	429	151	278	0.0762
Present	80	20	60	
pT stage				
T2	85	33	52	0.5263
T3	151	50	101	
T4	273	88	185	
pN stage				
N0	162	59	103	0.4947
N1	96	35	61	
N2	112	37	75	
N3	139	40	99	
TNM stage				
I	49	17	32	0.2255
II	172	66	106	
III	288	88	200	

^∗∗^ *P* < 0.01.

**Table 2:** Univariate analysis of prognostic parameters for survival in GC patients.

Prognostic parameter	HR	95% CI	*P* value
PD-L1 (positive versus negative)	0.668	0.505–0.885	0.005
PD-1 (high versus low)	0.767	0.495–1.189	0.236
CD3 (high versus low)	0.782	0.608–1.008	0.057
CD8 (high versus low)	0.691	0.536–0.891	0.004
FOXP3 (high versus low)	1.434	1.061–1.938	0.019
Age (>60 versus ≤60)	1.057	0.820–1.363	0.668
Gender (male versus female)	0.850	0.652–1.109	0.230
Tumor location (low versus others)	0.828	0.644–1.066	0.144
Length-diameter (≥5 cm versus <5 cm)	2.012	1.542–2.624	0.000
Lauren type (diffuse versus intestinal mix)	1.340	1.023–1.753	0.033
		
Perineuronal invasion (absent versus present)	0.578	0.419–0.796	0.001
		
Blood vessel invasion (absent versus present)	0.500	0.372–0.671	0.000
		
TNM stage (I–II versus III)	0.291	0.216–0.390	0.000

HR: hazard ratio; CI: confidence interval.

## Data Availability

The TCGA database analyzed during the current study is available in The Cancer Genome Atlas website (https://cancergenome.nih.gov/). Other data used to support the findings of this study can be requested from the corresponding author by email (zhaogang@renji.com).

## Supplementary Materials

Supplementary Materials Supplementary Table S1: correlation between PD-1 expression and clinicopathological characteristics in AGC patients. Supplementary Table S2: correlation between CD3 expression and clinicopathological characteristics in AGC patients. Supplementary Table S3: correlation between CD8 expression and clinicopathological characteristics in AGC patients. Supplementary Table S4: correlation between FOXP3 expression and clinicopathological characteristics in AGC patients.
