# Supplementary material for: PD-L1 Expression and CD8+ T Cell Infiltration Predict a Favorable Prognosis in Advanced Gastric Cancer
Source: J Immunol Res. 2018 May 29;2018:4180517. doi: 10.1155/2018/4180517 (PMC5996418; doi:10.1155/2018/4180517)
Supplement: Supplementary Materials — Supplementary Table S1: correlation between PD-1 expression and clinicopathological characteristics in AGC patients. Supplementary Table S2: correlation between CD3 expression and clinicopathological characteristics in AGC patients. Supplementary Table S3: correlation between CD8 expression and clinicopathological characteristics in AGC patients. Supplementary Table S4: correlation between FOXP3 expression and clinicopathological characteristics in AGC patients. [file 4180517.f1.pdf]

Supplementary Table S1: Correlation between PD-1 expression and clinicopathological characteristics in AGC patients.

Clinicopathological features	Cases	PD-1 expression		P-value(χ2 test)
		High	Low	
		58	451	
**Gender**				
Male	347	43	304	0.3002
Female	162	15	147	
**Age(years)**				
≤60	224	25	199	0.8828
> 60	285	33	252	
**Tumor location**				
Up	73	8	65	0.5121
Middle	105	8	97	
Low	260	35	225	
Total	66	7	59	
Remnant	5	0	5	
**Length-diameter**				
< 5cm	232	22	210	0.2141
≥5cm	277	36	241	
**Lauren type**				
Intestinal	163	13	150	0.2392
Diffuse	326	42	284	
Mix	20	3	17	
**Blood-vessel invasion**				
absent	413	43	370	0.1476
present	96	15	81	
**Perineuronal invasion**				
absent	429	43	386	0.0241*
present	80	15	65	
**pT stage**				
T2	85	10	75	0.8254
T3	151	19	132	
T4	273	29	244	
**pN stage**				
N0	162	17	145	0.9415
N1	96	12	84	
N2	112	12	100	
N3	139	17	122	
**TNM stage**				
I	49	4	45	0.7542
II	172	20	152	
III	288	34	254	

* : $P<0.05$, ** : $P<0.01$;

Supplementary Table S2: Correlation between CD3 expression and clinicopathological characteristics in AGC patients.

Clinicopathological features	Cases	CD3 expression		*P*-value(χ2 test)
		High	Low	
		254	255	
**Gender**				
Male	347	173	174	0.9758
Female	162	81	81	
**Age(years)**				
≤60	224	121	103	0.0996
> 60	285	133	152	
**Tumor location**				
Up	73	41	32	0.2202
Middle	105	43	62	
Low	260	131	129	
Total	66	37	29	
Remnant	5	2	3	
**Length-diameter**				
< 5cm	232	117	115	0.8907
≥5cm	277	138	139	
**Lauren type**				
Intestinal	163	67	96	0.0243*
Diffuse	326	176	150	
Mix	20	11	9	
**Blood-vessel invasion**				
absent	413	206	207	0.9830
present	96	48	48	
**Perineuronal invasion**				
absent	429	214	215	0.9847
present	80	40	40	
**pT stage**				
T2	85	41	44	0.9445
T3	151	76	75	
T4	273	137	136	
**pN stage**				
N0	162	75	87	0.3927
N1	96	55	41	
N2	112	56	56	
N3	139	68	71	
**TNM stage**				
I	49	21	28	0.5837
II	172	87	85	
III	288	146	142	

*：*P*<0.05，**：*P*<0.01；

Supplementary Table S3: Correlation between CD8 expression and clinicopathological characteristics in AGC patients.

Clinicopathological features	Cases	CD8 expression		P-value(χ2 test)
		High	Low	
		254	255	
**Gender**				
Male	347	173	174	0.9758
Female	162	81	81	
**Age(years)**				
≤60	224	114	110	0.6918
> 60	285	140	145	
**Tumor location**				
Up	73	44	29	0.0859
Middle	105	45	60	
Low	260	124	136	
Total	66	39	27	
Remnant	5	2	3	
**Length-diameter**				
< 5cm	232	112	120	0.5020
≥5cm	277	142	135	
**Lauren type**				
Intestinal	163	71	92	0.1171
Diffuse	326	171	155	
Mix	20	12	8	
**Blood-vessel invasion**				
absent	413	211	202	0.2663
present	96	43	53	
**Perineuronal invasion**				
absent	429	211	218	0.4534
present	80	43	37	
**pT stage**				
T2	85	36	49	0.3008
T3	151	79	72	
T4	273	139	134	
**pN stage**				
N0	162	84	78	0.0242*
N1	96	59	37	
N2	112	46	66	
N3	139	65	74	
**TNM stage**				
I	49	21	28	0.3073
II	172	93	79	
III	288	140	148	

* : *P*<0.05 , ** : *P*<0.01 ;

Supplementary Table S4: Correlation between FOXP3 expression and clinicopathological characteristics in AGC patients.

Clinicopathological features	Cases	FOXP3 expression		P-value(χ2 test)
		High	Low	
		97	412	
**Gender**				
Male	347	71	276	0.2378
Female	162	26	136	
**Age(years)**				
≤60	224	38	186	0.2865
> 60	285	59	226	
**Tumor location**				
Up	73	10	63	0.3760
Middle	105	19	86	
Low	260	57	203	
Total	66	11	55	
Remnant	5	0	5	
**Length-diameter**				
< 5cm	232	49	183	0.2780
≥5cm	277	48	229	
**Lauren type**				
Intestinal	163	26	137	0.3831
Diffuse	326	68	258	
Mix	20	3	17	
**Blood-vessel invasion**				
absent	413	80	333	0.7088
present	96	17	79	
**Perineuronal invasion**				
absent	429	82	347	0.9393
present	80	15	65	
**pT stage**				
T2	85	17	68	0.9626
T3	151	29	122	
T4	273	51	222	
**pN stage**				
N0	162	35	127	0.8005
N1	96	17	79	
N2	112	20	92	
N3	139	25	114	
**TNM stage**				
I	49	12	37	0.5844
II	172	31	141	
III	288	54	234	

* : P<0.05 , ** : P<0.01 ;
